# A novel non-invasive method allowing for discovery of pathologically relevant proteins from small airways

**DOI:** 10.1186/s12014-022-09348-y

**Published:** 2022-06-06

**Authors:** Jörgen Östling, Marleen Van Geest, Henric K. Olsson, Sven-Erik Dahlen, Emilia Viklund, Per M. Gustafsson, Ekaterina Mirgorodskaya, Anna-Carin Olin

**Affiliations:** 1 Department of Bioscience, Respiratory, Inflammation and Autoimmunity, IMED Biotech Unit, AstraZeneca, Gothenburg, Sweden; 2 Translational Science and Experimental Medicine, Research and Early Development, Respiratory & Immunology, BioPharmaceuticals R&D, AstraZeneca, Gothenburg, Sweden; 3 Institute of Environmental Medicine, Karolinska Institute, Stockholm, Sweden; 4 Occupational and Environmental Medicine, Department of Public Health and Community Medicine, Institute of Medicine, Sahlgrenska Academy, University of Gothenburg, Gothenburg, Sweden; 5 Department of Paediatrics, Central Hospital, Skövde, Sweden; 6 Core Facilities, Sahlgrenska Academy, University of Gothenburg, Gothenburg, Sweden; 7 PExA AB, Gothenburg, Sweden; 8 Hansa Biopharma AB, Lund, Sweden

**Keywords:** Exhaled air, Non-volatiles, Breath, Small airways, Biomarker, Non-invasive, Asthma, Proteomics, Precision medicine

## Abstract

**Background:**

There is a lack of early and precise biomarkers for personalized respiratory medicine. Breath contains an aerosol of droplet particles, which are formed from the epithelial lining fluid when the small airways close and re-open during inhalation succeeding a full expiration. These particles can be collected by impaction using the PExA method (Particles in Exhaled Air), and are derived from an area of high clinical interest previously difficult to access, making them a potential source of biomarkers reflecting pathological processes in the small airways.

**Research question:**

Our aim was to investigate if PExA method is useful for discovery of biomarkers that reflect pathology of small airways.

**Methods and analysis:**

Ten healthy controls and 20 subjects with asthma, of whom 10 with small airway involvement as indicated by a high lung clearance index (LCI ≥ 2.9 z-score), were examined in a cross-sectional design, using the PExA instrument. The samples were analysed with the SOMAscan proteomics platform (SomaLogic Inc.).

**Results:**

Two hundred-seven proteins were detected in up to 80% of the samples. Nine proteins showed differential abundance in subjects with asthma and high LCI as compared to healthy controls. Two of these were less abundant (ALDOA4, C4), and seven more abundant (FIGF, SERPINA1, CD93, CCL18, F10, IgM, IL1RAP). sRAGE levels were lower in ex-smokers (n = 14) than in never smokers (n = 16). Gene Ontology (GO) annotation database analyses revealed that the PEx proteome is enriched in extracellular proteins associated with extracellular exosome-vesicles and innate immunity.

**Conclusion:**

The applied analytical method was reproducible and allowed identification of pathologically interesting proteins in PEx samples from asthmatic subjects with high LCI. The results suggest that PEx based proteomics is a novel and promising approach to study respiratory diseases with small airway involvement.

**Supplementary Information:**

The online version contains supplementary material available at 10.1186/s12014-022-09348-y.

## Key messages

**Key question:**

Can the PExA method identify individual protein profiles that reflect pathology of small airways, using the SOMAscan platform?

**What is the bottom line?:**

Two hundred-seven proteins were detected in up to 80% of the PEx samples, with a strong overrepresentation of proteins related to innate immune responses, including nine proteins that that discriminated subjects with asthma and high LCI as compared to healthy controls.

**Why read on:**

The results support that PEx based proteomics is a novel and promising approach to study respiratory diseases with small airway involvement.

**Supplementary Information:**

The online version contains supplementary material available at 10.1186/s12014-022-09348-y.

## Introduction

There is a growing interest in the role of small airways (inner diameter < 2 mm) in asthma and other lung diseases [1]. In asthma, involvement of small airways is associated with more severe disease and loss of control [2–6], but has also been demonstrated in moderate and mild asthma [7]. Small airway involvement is also a recognised feature of chronic obstructive lung disease (COPD) [8], and in other severe lung-disease including viral bronchiolitis (as observed in e.g., COVID-19), lung-fibrosis and hypersensitivity pneumonitis.

In the small airways, surfactant plays a crucial role for airway patency and innate immune responses [9]. Surfactant is a complex mixture of proteins and lipids that keeps small airways open by reducing surface tension, but it also plays an important role in innate immunity by enhancing phagocytosis of inhaled pathogens and particulate matter by special surfactant proteins [10] and by modulating immune responses [11–14]. Given its crucial role for airway patency and host defence, knowledge of the protein and lipid composition of surfactant is surprisingly limited.

Although the small airways are a key compartment for the onset and progression of respiratory diseases such as COPD and lung-fibrosis [15], early detection of pathological processes in the small airways remains difficult mainly due its inaccessibility. One option for retrieving biological material from the small airways is through bronchoscopy with sampling of biopsies or bronchoalveolar lavage fluid (BALF), but this method is invasive and not suited for point-of-care situations or clinical trials. Non-invasive physiological measurements reflecting small airway function exist (e.g., inert-gas washout techniques and impulse oscillometry), but these methods do not provide the molecular information about pathways needed for the further development of precision medicine. In particular, the introduction of biologics, targeting specific molecular pathways, have highlighted the need for biomarkers that reflect disease endotypes, to enable patient stratification.

Particles in Exhaled Air (PExA) is a novel sampling method allowing non-invasive retrieval of biological material from the small airways. In short, the method is based on impaction of an aerosol consisting of ultrafine droplets of respiratory tract lining fluid (RTLF) that are formed and exhaled after a breathing manoeuvre that promote airway closure and reopening of the small airways [16]. PExA method has been thoroughly described by Larsson et al. [17].

The molecular composition of PEx samples have been explored in previous studies, and 120 different proteins could be detected in PEx samples pooled from several individuals by LC/MS [20]. The protein composition of these samples showed up to 80% similarities to BALF.

Highly abundant proteins, like Surfactant protein A (SP-A) have been successfully quantified with low intra-individual variability in PEx samples from single individuals by ELISA [17, 18] and show good correlation to SP-A levels in BALF [19]. The small airway origin of the PEx sample is supported both by its composition, resembling (BALF) but not bronchial wash (BW) [19], and that no amylase is detected by LC/MS [20]. It is also indirectly supported by the 1000–10,000 fold increase in number of exhaled and sampled particles when using a breathing manoeuvre that promote airway closure and re-opening [16, 21].

In the present study we sought to evaluate whether PEx samples convey information on pathophysiological processes useful in biomarker discovery. SOMAscan (SomaLogic Inc.) was identified as a potentially suitable proteomics platform for the study. As PEx samples mainly originate from the small airways, we hypothesised that differences in protein composition of PEx samples would be easiest to observe studying patients with increased lung clearance index (LCI), a standard measure of global ventilation inhomogeneity, i.e., an indirect measure of small airway involvement that also is considered a sensitive indicator of early lung damage. Based on this reasoning we chose to study the protein composition of samples from asthmatic subjects with high LCI compared to that of asthmatic subjects and heathy controls with normal LCI.

## Methods and analysis

At first, we evaluated the performance of the SOMAscan platform and reproducibility. The second step was a clinical evaluation in a cross-sectional design, where the pathological relevance and the differences in protein-profiles in PEx of non-asthmatic subjects with that of subjects with asthma with- or without high LCI, were assessed.

### Subjects

Twenty subjects with asthma and ten healthy controls were included in the clinical evaluation, all of Caucasians origin. All were recruited from our earlier studies on asthma, or by an advertisement in a daily paper. To identify subjects with small airway involvement all subjects were screened with multiple breath nitrogen wash test (MBNW), giving an index of heterogeneity of ventilation (LCI). Asthma subjects were stratified into two groups, whereof one with normal LCI (z-score < 2), herein referred to as A-nLCI (n = 10), and one with high LCI (z-score ≥ 2.9), herein referred to as A-hLCI (n = 10). All subjects with asthma reported a physician diagnose of asthma and were taking asthma medication regularly. We also included a control group (non-asthma) that did not report respiratory symptoms nor were taking medication for respiratory disease and had normal LCI z-score (i.e. LCI < 2), herein referred to as NA (n = 10)[22].

Exclusion criteria were current smoking or smoking within the last 10 years or > 10 pack-years, diagnosis of systemic inflammatory disease, cardiovascular disease or pregnancy. Demographic and clinical data including LCI z-scores are presented in Table 1. All participants gave their written informed consent and the study was approved by the Ethical Committee at Gothenburg University in Sweden.

**Table 1:** Demographic and clinical characteristics of the three study-groups, including result from statistical tests

Parameter	Non-asthma (NA)	Asthma with normal LCI (A-nLCI)	Asthma with high LCI (A-hLCI)	p values		
				A-hLCI vs. NA	A-hLCI vs. A-nLCI	A-nLCI vs. NA
Number	10	10	10			
Gender (male/female)	7/3	4/6	3/7	ns	ns	ns
Age	48.9 (4.43) [28–66]	38.1 (4.1) [20–59]	54.6 (3.23) [38–68]	ns	0.0040	ns
Age at onset of asthma, yrs	–	17.4 (5.16) [5–55]	24.3 (7.38) [2.0–60]	–	ns	–
BMI	23.89 (0.77) [19.26–27.16]	24.24 (0.8) [21.15–28.34]	25.93 (0.97) [21.47–31.18]	ns	ns	ns
Allergy y/n	3/7	7/3	6/4	ns	ns	ns
Ex smoker y/n	3/7	4/6	7/3	ns	ns	ns
FEV1 (% pred)	100.9 (2.9) [88–117]	93.6 (4.63) [79–123]	71.2 (5.31) [39–91]	0.0003	0.0051	ns
FEV1/FVC (%)	75.13 (7.67) [7.77–93.8]	79.2 (2.11) [71–88]	62.69 (3.73) [35–71]	0.0024	0.0001	ns
Reversibility (%)	2.4 (1.66) [− 5–9]	7.5 (2.31) [1–21]	14.7 (2.68) [6–28]	0.0006	0.0137	ns
LCI z-score	0.89 (0.12) [0.5–1.7]	1.04 (0.17) [0–1.8]	5.07 (0.53) [2.9–8.1]	0.0001	0.0001	ns
S-Cond VT, z-score	− 0.57 (0.42) [− 3–1.7]	− 0.13 (0.4) [− 1.5–1.4]	3.77 (0.49) [0.7–5.8]	0.0002	0.0005	ns
S-Acin VT, z-score	0.59 (0.18) [0–1.5]	0.48 (0.21) [0–1.8]	2.76 (0.81) [0–8.6]	0.0077	0.0065	ns
GINA step	–	2.2 (0.29) [1–4]	2.9 (0.41) [1–4]	–	ns	–
ACQ, mean (1–6)	–	0.82 (0.3) [0–3.17]	1.13 (0.24) [0–2.17]	–	ns	–
B-neutrophils (%)	3.01 (0.33) [1.5–4.4]	3.68 (0.35) [2.2–5.4]	4.53 (0.52) [2.5–7.3]	0.0493	ns	ns
B-eosinophils(%)	0.15 (0.04) [0.06–0.5]	0.27 (0.04) [0.1–0.6]	0.3 (0.07) [0.1–0.6]	0.0287	ns	0.0156
FENO, ppb	17.7 (1.93) [8–24]	41.2 (8.72) [6–86]	41.7 (11.74) [11–113]	ns	ns	ns
hsCRP	0.51 (0.08) [0.23–1.1] (n = 9)	0.432 (0.107) [0.14–1.2]	2.35 (0.62) [0.45–5.40]	0.0054	0.0031	ns
Average mass pg/particle	0.23 (0.01) [0.2–0.27]	0.22 (0.01) [0.18–0.3]	0.22 (0.01) [0.17–0.29]	ns	ns	ns

Data are presented as, means with standard error given in parenthesis and range given in brackets. Incomplete data is indicated by n numbers given in parenthesis. Kruskal Wallis and Chi Square statistical tests were used for analysing the differences between continuous and categorical data, respectively. “ns” indicate statistical test with p value below 0.05. Dash (–) indicate “not applicable”

### Clinical characterization

Spirometry was performed according to ERS guidelines, using Spirare spirometer (Spirare, Stockholm, Sweden) Forced vital capacity (FVC) and forced expired volume in one second were expressed as a percentage of the reference value (FEV1% pred) derived from the ECCS/ERS reference equations [23].

Multiple Breath Nitrogen Wash-out tests were performed using the Exhalyzer^®^ D device (Eco Medics AG, Duernten, Switzerland) and software (Spiroware 3.1) in accordance with current guidelines [24]. Z-scores were calculated as described by Kjellberg et al. [25].

Fraction of exhaled nitric oxide (FENO) was measured once by a NIOX Mino (Aerocrine AB, Stockholm, Sweden) before spirometry following the ATS-ERS guidelines [26], except for only performing one exhalation.

A skin-prick test (SPT) to common allergens in Sweden was performed with positive result defined as a wheal diameter ≥ 3 mm and negative control < 3 mm. Atopy was defined as the occurrence of at least one positive SPT wheal.

Serum samples were analysed for hsCRP and differential cell counts, using standard clinical methods.

All subjects filled out a questionnaire on medical history, smoking habits, symptoms and medication and subjects with asthma filled out Asthma Control Questionnaire, ACQ, reflecting asthma control over the last week [27]. The use of medication was translated to GINA step for each subject according to GINA guidelines 2016.

### PEx sample collection

The PExA method and PExA 1.0 instrument was used to collect PEx samples (described in supplement). For assessment of reproducibility, 120 ng of PEx was collected from each subject and for all other samples at least 240 ng of PEx was collected, involving two consecutive sampling sessions with a short break in between. After collection the sample holder was transferred to a clean-air room and the substrate was excised with a scalpel from the sample holder and placed in Millipore Ultrafree-MC LH Centrifugal Filter insert (FC30LH25) and stored at − 80 °C for further analysis. True blank samples were generated by applying the same procedure as for real samples but without a human breathing into the PExA instrument.

### SOMAscan analysis and data processing

SOMAscan is a proprietary highly multiplexed, sensitive proteomic platform (SomaLogic Inc., Boulder, USA). As the SOMAscan platform developed during the study period two different versions was used; (i) SOMAscan 1.1 K was used for the assessment of SOMAscan performance with PEx samples and SOMAscan 1.3 K for the other experiments. Platform and sample preparation is described in supplement.

Intra-run normalization and inter-run calibration were performed by SomaLogic according to their SOMAscan assay GLP data quality-control procedures. Data from SomaLogic was reported in relative fluorescent units (RFU) after hybridization control normalization which remove individual sample variance on the basis of signalling differences between scans (herein referred to as RFU values). Data from all samples passed quality-control criteria and were considered eligible for further analysis. Limit of detection (LOD) was calculated as 3 times the standard deviation from the mean RFU signal measured from 3 blank samples. Proteins with RFU values below LOD were not considered for further analyses. To account for systematic differences due to possible variability in final PEx concentration, the set of detected proteins were subjected to group median based normalization and log2 transformation before statistical analysis was performed. Mean and median values for establishment of LOD and normalization, respectively were calculated based on RFU values in all samples.

### Gene Ontology enrichment analysis

To improve our understanding of the origin and functions of the proteins seen in PEx samples, a protein annotation enrichment analysis was performed, using the publicly available “Gene Ontology enrichment analysis and visualization tool GOrilla [28], matching a list of 199 uniquely mapped PEx proteins to either the Cellular Component (CC) or the Biological Process (BP) GO sub-domain (database updated on Feb 15, 2020). A list of 1291 uniquely mapped SOMAscan protein identities was used as reference/background.

### Statistical analysis

Significance level for the Gene Ontology enrichment analysis was calculated using the right-tailed Fisher exact test, provided by the GOrilla web-based service [28]. Result from GO annotation enrichment analysis were considered significant at a Benjamini–Hochberg corrected p value below 0.05. PEx protein composition was compared to that of BAL and enrichment factor was calculated by Fisher Exact test.

SOMAscan data were mainly analysed using Qlucore Omics Explorer 3.6 software (Qlucore, Lund, Sweden). RFU values for the 207 proteins was found to not meet requirements for normality and was therefore log2 transformed before statistical analysis. One-way analysis of variance (ANOVA) tests were used to determine intra-individual differences in the reproducibility experiment. General linear model statistics with each variable normalized to mean 0 and variance of 1 and adjustment for imbalance in age and BMI, was used to test differences between the NA, A-nLCI and A-hLCI groups. Benjamini–Hochberg multiple correction was used to control for rate of false-positive results (herein referred to as q value). Statistical analysis of clinical and demographic variables was performed with Kruskal–Wallis or Chi-square tests using Spotfire 7.0.2 software (TIBCO Spotfire).

Group comparisons of SOMAscan data were considered hypothesis free and proteins with p value below 0.05 and a q value below 0.2 was considered to be of interest in this explorative study.

## Results

### Assessment of SOMAscan assay performance for PEx samples

**Fig. 1:**
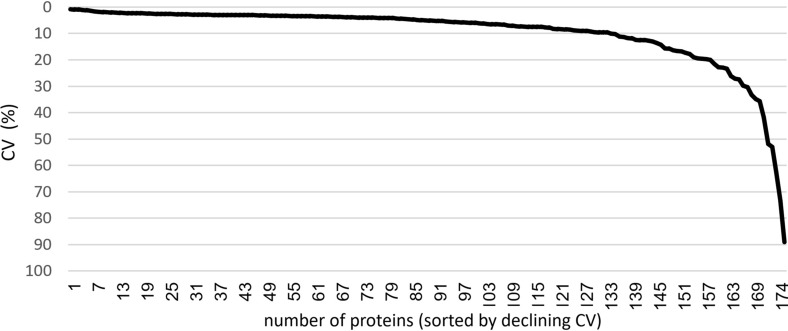
Distribution of CV for 174 proteins detected in a pooled PEx sample (1 µg PEx/ml), analysed 5 times with the SOMAscan 1.1 K platform. The pooled sample originated from 6 subjects with asthma and 3 healthy volunteers. Proteins were considered detected if RFU values delivered by SomaLogic were larger than LOD in all 5 replicate samples. Limit of detection (LOD) was calculated as 3 times the standard deviation from the mean RFU signal measured from 3 blank samples

**Fig. 2:**
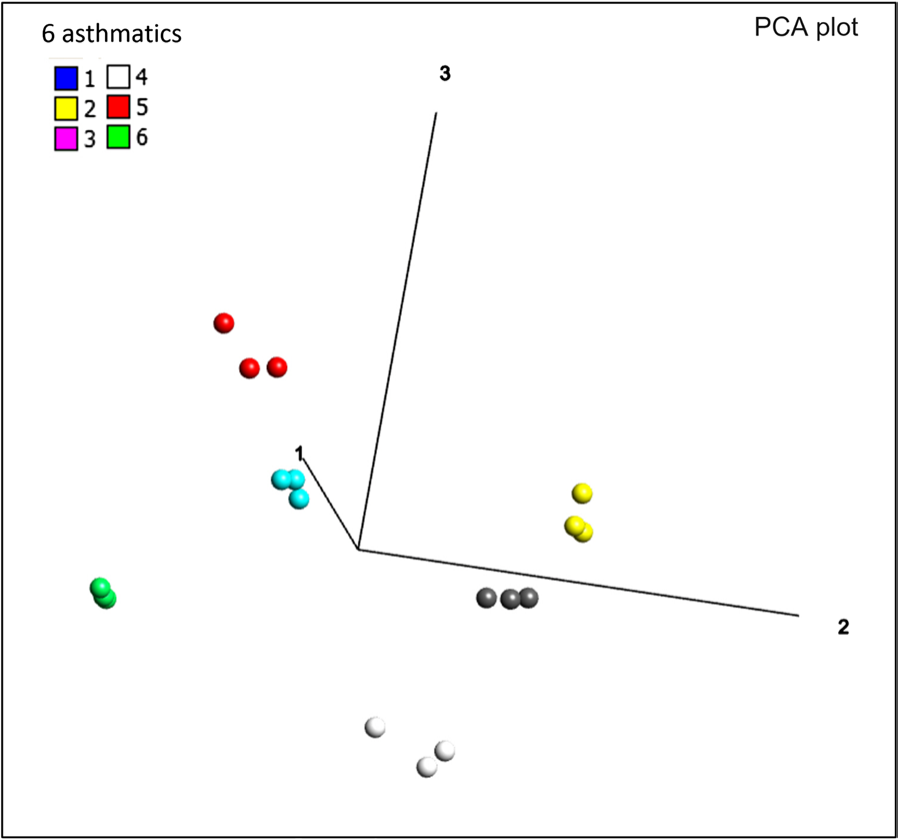
Assessment of intra-individual variability by visual inspection of Principle Component Analysis (PCA) plot. PEx samples from 3 consecutive PEx samples from 6 asthmatic subjects (red, blue, green, white, black and yellow) were analysed with the SOMAscan 1.1 K platform. Using ANOVA statistical test based variable selection (q < 5.5E−5) 42 out of 114 proteins commonly detected in all 18 samples, were found to discriminate all 6 subjects from each other in a PCA plot, as judged by visual inspection in Omics Explorer software

SOMAscan technical variability was evaluated by repeated measurements of a pooled PEx sample (1 µg PEx per ml) 5 times on the SOMAscan 1.1 K platform. The mean CV value was 10% looking at a set of 174 proteins detected in all five samples, and below 20% for 156 of the 174 detected proteins (Fig. 1). Intra-individual repeatability related to the PEx sampling procedure and the SOMAscan 1.1 K platform combined, was evaluated by repeat measurements of three consecutive 120 ng PEx samples collected from 6 subjects with asthma. The intra individual CV values ranged from 6.1 to 24.8% with a mean of 13.8%, looking at a set of 114 proteins detected in all 18 samples. To assess if the observed intra-individual variability is low enough for the method to be useful for biomarker discovery, we analysed to what degree it was possible to separate the 6 subjects from each other, solely based on the proteomics data. Defining each of the 6 triplicate samples as groups, the between groups ANOVA test revealed 102 proteins with statistically significant differences between at least two of the group means (q < 0.05). Filtering the list of protein variables further down to a q value cut-off of 5.5 × 10^–5^ yielded 42 proteins that completely separated all 6 subjects from each other, as judged by visual inspection of a Principal Component Analysis (PCA) plot (Fig. 2). This means that the intra-individual variation was very low compared to the inter-individual variation, when comparing three samples from each individual sampled on the same day.

### Assessment of pathological relevance of proteins detected in PEx samples

Of the 1310 proteins represented on the 1.3 K SOMAscan panel, 134 proteins showed RFU values larger than LOD in the complete set of 30 samples (2 µg PEx/ml). To increase chance of finding differentially abundant proteins a set of 207 SOMAscan protein ID’s, detected over LOD in 80% of the 30 samples (Additional file 1: Table S1) were used for various comparative data analyses.

#### Comparison of the protein composition of PEx with that of BALF by enrichment analysis

Of 207 proteins detected with the SOMAscan 1.3 K platform, 81 (41%) have previously been detected in BALF [29]. Using 1323 uniquely mapped SOMAscan protein identities as reference/background gave at hand that the 207 proteins detected in PEx samples are enriched 5.9 times with the proteins previously detected in supernatant from BALF samples (p < 0.0001).

#### Gene Ontology (GO) enrichment analysis

**Fig. 3:**
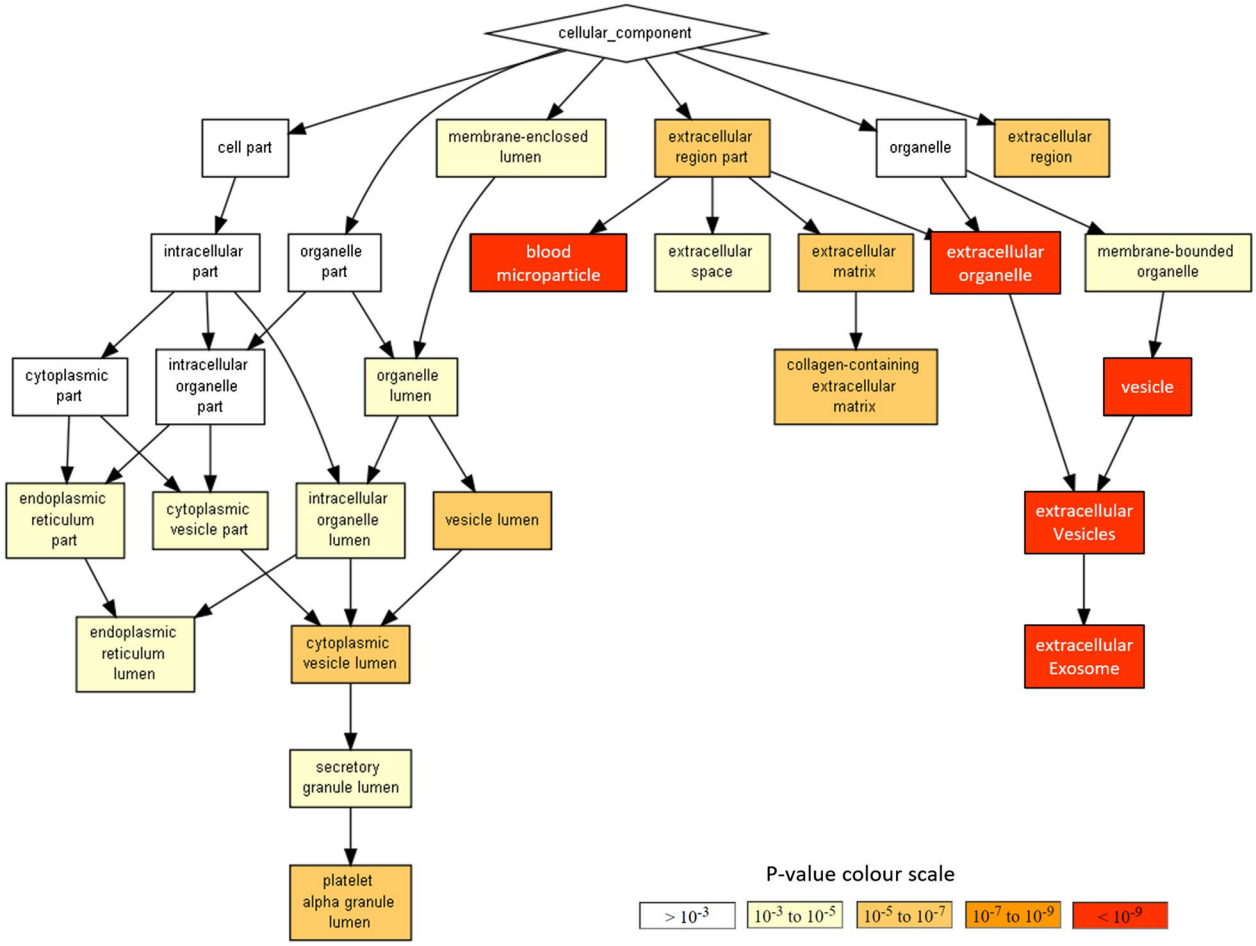
Visualization of results from Gene Ontology (GO) enrichment analysis (GOrilla [28]) matching 207 proteins detected in PEx samples by SOMAscan 1.3 K platform, to the GO Cellular Component sub-domain database. Over represented GO terms are organized in a parent–child based hierarchically structure with color-coded significance levels (Fisher’s exact test), as indicated in the p value colour scale

Gene Ontology enrichment analysis of 199 uniquely mapped PEx/SOMAscan protein ID’s (Fig. 3) revealed an over-representation of several Cellular Components (CC) GO terms, for example; “extra cellular exosome” [enrichment factor (EF) = 1.79, q = 6.30E−11], “blood microparticle” (EF = 3.43, q = 8.28E−10) and “platelet alpha granule lumen” (EF = 3.15, q = 1.78E−04) (Table 2). Biological Process (BP) GO domain analysis revealed an over-representation of BP terms, for example; “regulation of complement activation” (EF = 4.4, q = 5.17E−08), “platelet degranulation” (EF = 2.8, q = 2.88E−04), “regulation of coagulation” (EF = 2.6, q = 2.72E−02), “acute inflammatory response” (EF = 3.21, q = 8.08E−03), and “neutrophil activation involved in immune response” (EF = 1.69, q = 2.9E−02) (Table 2B).

**Table 2:** Gene ontology annotation enrichment analysis

Section A: Cellular Component sub-domain (CC)					
Description (term)	Enrichment factor	Input and output N,B,n,b	p value	FDR q value	GO term
Extracellular exosome	1.79	1291,382,194,103	6.80E−14	6.30E−11	GO:0070062
Extracellular vesicle	1.78	1291,384,194,103	1.03E−13	3.19E−11	GO:1903561
Extracellular region	1.39	1291,566,194,118	1.92E−07	2.97E−05	GO:0005576
Blood microparticle	3.43	1291,62,194,32	3.57E−12	8.28E−10	GO:0072562
Platelet alpha granule lumen	3.15	1291,38,194,18	1.53E−06	1.78E−04	GO:0031093
Extracellular matrix	1.82	1291,183,194,50	2.12E−06	2.18E−04	GO:0031012
Collagen-containing extracellular matrix	1.91	1291,150,194,43	3.35E−06	3.11E−04	GO:0062023
Cytoplasmic vesicle lumen	1.92	1291,135,194,39	8.75E−06	6.76E−04	GO:0060205
Endoplasmic reticulum lumen	1.97	1291,91,194,27	1.61E−04	8.27E−03	GO:0005788
Endoplasmic reticulum part	1.71	1291,132,194,34	4.70E−04	2.18E−02	GO:0044432

Section B: Biological Process sub-domain (BP)					
Description (term)	Enrichment factor	Input and output N,B,n,b	p value	FDR q value	GO term
Regulation of extracellular matrix constituent secretion	6.65	1291,5,194,5	7.33E−05	2.23E−02	GO:0003330
Exocytosis	1.80	1291,185,194,50	3.05E−06	1.54E−03	GO:0006887
Vesicle-mediated transport	1.74	1291,276,194,72	3.05E−08	2.78E−05	GO:0016192
Secretion by cell	1.63	1291,224,194,55	2.27E−05	8.63E−03	GO:0032940
Regulation of protein activation cascade	4.50	1291,34,194,23	2.28E−12	2.08E−08	GO:2000257
Regulation of complement activation	4.44	1291,33,194,22	1.13E−11	5.17E−08	GO:0030449
Complement activation, alternative pathway	4.44	1291,12,194,8	6.55E−05	2.14E−02	GO:0006957
Complement activation, classical pathway	3.90	1291,29,194,17	5.25E−08	4.36E−05	GO:0006958
Regulation of humoral immune response	3.64	1291,42,194,23	1.17E−09	1.77E−06	GO:0002920
Innate immune response	1.81	1291,114,194,31	3.04E−04	6.03E−02	GO:0045087
Platelet degranulation	2.80	1291,57,194,24	3.78E−07	2.88E−04	GO:0002576
Fibrinolysis	3.52	1291,17,194,9	2.67E−04	5.41E−02	GO:0042730
Negative regulation of coagulation	3.21	1291,29,194,14	1.86E−05	7.37E−03	GO:0050819
Regulation of coagulation	2.60	1291,41,194,16	1.19E−04	2.72E−02	GO:0050818
Regulation of haemostasis	2.60	1291,41,194,16	1.19E−04	2.86E−02	GO:1900046
Acute-phase response	3.33	1291,24,194,12	4.93E−05	1.67E−02	GO:0006953
Acute inflammatory response	3.21	1291,29,194,14	1.86E−05	8.08E−03	GO:0002526
Neutrophil activation involved in immune response	1.69	1291,126,194,32	9.28E−04	1.69E−01	GO:0002283
Regulation of response to external stimulus	1.48	1291,287,194,64	1.14E−04	2.90E−02	GO:0032101
Defence response	1.40	1291,300,194,63	8.92E−04	1.70E−01	GO:0006952

This table display result from Gene Ontology enrichment analysis using the publicly available “Gene Ontology enrichment analysis and visualization tool” (GOrilla) [28]. A list of 199 uniquely mapped PEx proteins detected with SOMAscan 1.3 K were searched against the Cellular Component sub-domain database (section A) and the Biological Process sub-domain database (section B). A list of 1291 uniquely mapped SOMAscan 1.3 K protein identities was used as reference/background. Enrichment factor was calculated as (b/n)/(B/N), where n—is the total number of PEx protein ID’s, identified by SOMAscan and used as input, b—is the number of PEx/SOMAscan protein ID’s associated with the GO term. p values for enrichment analysis were computed according to the mHG or HG model. FDR q value is the p value corrected for multiple testing using the Benjamini and Hochberg (1995) method

#### Differential abundance analysis, asthma vs. non-asthma

**Fig. 4:**
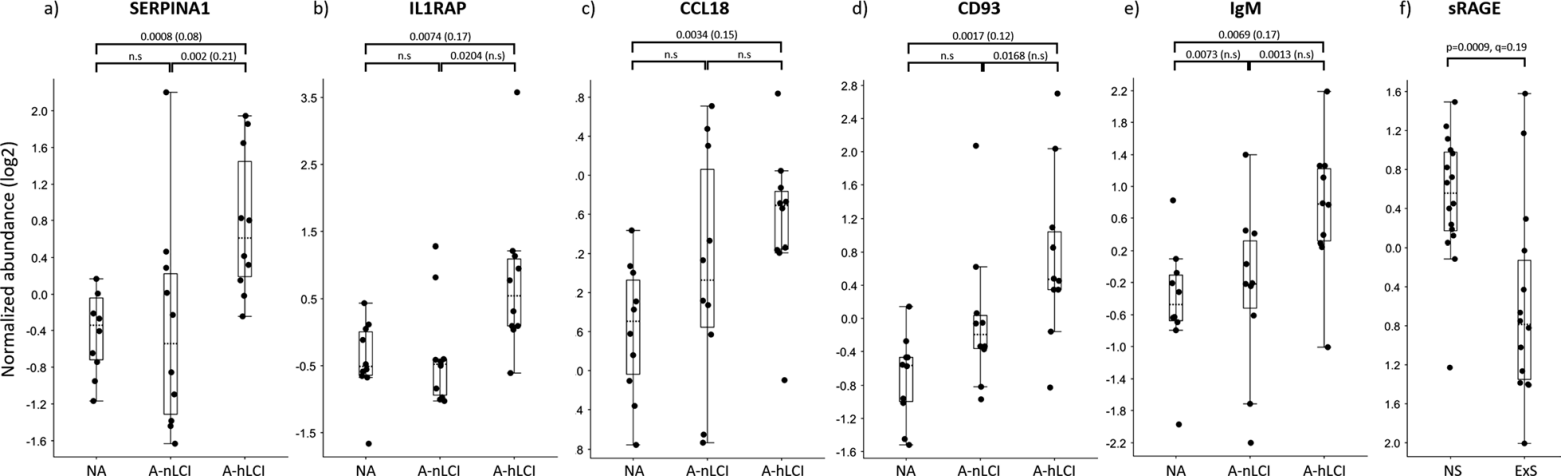
Box plots show examples of SOMAscan data for 6 differentially abundant proteins; **a** Alpha-1-antitrypsin (SERPINA1), **b** Interleukin-1 Receptor accessory protein (IL1RAP), **c** CC motif chemokine 18 (CCL18), **d** Complement component C1q receptor (CD93), **e** Immunoglobulin M (IgM), in non-asthma (NA), asthma without (A) and with small airway involvement (A-hLCI), and **f** Soluble Receptor of Advanced Glycation End products (sRAGE) in never-smokers (NS) and ex-smokers (ExS). Y-axis show normalized abundance (log2 transformation and normalization to mean 0 and variance 1). Box ranges from the 25th to the 75th percentile and median value is marked with dotted line. p values and false discovery rate adjusted p values (q) from various pairwise comparisons are shown over each box plot. Protein abundance data was adjusted for difference in age

To identify confounding demographic factors we investigated the impact of gender, BMI and age, and found a clear effect of age and to some extent of BMI, independent of disease status. The relative abundance of each of the 207 detected proteins were then compared between various pairwise combinations of the A-hLCI, (n = 10), A-nLCI (n = 10) and NA (n = 10) groups. Adjusting for imbalance in age, 9 proteins were found to be differentially abundant in A-hLCI as compared to the NA group, whereof 2 were less abundant (ALDOA4, C4) and 7 more abundant in A-hLCI (FIGF, SERPINA1, CD93, CCL18, F10, IgM, IL1RAP) (Additional file 1: Table S2), exemplified in Fig. 4. Reviewing the scientific literature revealed that all of the 9 differentially abundant proteins are known to play role in immune response and respiratory disease (Additional file 1: Table S3).

#### Differential abundance analysis, ex-smokers vs. never smokers

To explore effect of smoking in a post-hoc analysis, the 207 SOMAscan/PEx protein data set was screened for proteins showing differential abundance in ex-smokers (n = 14) vs. never smokers (n = 16) (Additional file 1: Table S4). Only one protein, sRAGE (soluble Receptor for Advanced Glycation End products), a pattern-recognition receptor involved in host response to injury, infection and inflammation fulfilled the significance criteria after adjusting for age and BMI, with decreased abundance in ex-smokers as compared to never smokers (Fig. 4). By contrast, sRAGE did not show any clear difference between any of the asthma groups and healthy controls.

## Discussion

Exhalation after breath-holding at residual volume give rise to release of high numbers of tiny droplets/particles formed from the respiratory tract lining fluid covering the small airways. Some of these particles are small enough to follow the airstream of the exhalation and can be collected by impaction technology (PExA). Due to the small size of the particles and the specific origin, the total amount of respiratory tract lining fluid that can be collected in this way is minute. In the present study we addressed the feasibility of proteomic profiling of PEx samples and could demonstrate that the SOMAscan proteomics platform is sensitive enough to detect and accurately quantify over 150 proteins in PEx samples from single individuals. Given that the SOMAscan panel cover close to 1300 unique protein IDs, 150 may sound as a relative small number. However, since the SOMAscan platform have been developed primarily for analysis of blood samples and a PEx samples originate only from exhaled air and therefore only contain 100–200 ng of a undiluted body fluid, with an unknown dynamic range, we believe that detection of more than 150 proteins in the complete set of 30 samples is a surprisingly good result. Although, limited number of proteins was detected, one should bear in mind that all these proteins originate directly from the Small airways, a highly relevant region for respiratory research, which otherwise would have been very difficult to sample in a non-invasive way.

Analysis of three consecutive samples indicated that intra-individual variability is substantially smaller than the inter-individual variability.

Moreover, protein enrichment analysis showed that protein composition of the PEx matrix resembles that of BALF supernatant to a large extent. This finding provide further confidence and confirms previous findings that PEx sample originate from small airways [19, 20] and hold the potential to be developed into a non-invasive substitute for bronchoscopy based diagnosis.

Protein enrichment analysis revealing that the PEx matrix is enriched in extracellular proteins associated with “exosome” (Fig. 3, Table 2), is of particular interest due to the emerging role of the exosomes as mediators of biomarkers for several chronic lung diseases [30, 31]. In addition, PEx proteome seem highly relevant for studies on the role of innate immune response in development of respiratory diseases and host defence.

To explore the pathological relevance of the PEx proteome in studies of respiratory disease we analysed PEx/SOMAscan data from 20 asthma patients and 10 healthy control subjects. Despite the low number of subjects, we found several highly interesting proteins to be differently abundant in samples from subjects with asthma compared to the non-asthma group. Alfa-1-antitrypsin (SERPIN1A) and IL1RAP were elevated only in asthma patients with high LCI, as opposed to IgM, CD93 and CCL18 which were elevated also in asthma patients without small airway involvement (Fig. 4A). The two different profiles suggest that SERPIN1A and IL1RAP may be specifically involved in small airway dysfunction, whereas IgM, CD93 and CCL18 may reflect disease processes less specific for small airway pathology. The post-hoc analysis showed that level of sRAGE, a protein suggested to be a blood based biomarker of smoking induced pathology [32, 33] was found to be lower in PEx from ex-smokers, suggest that PEx samples is capable of reflecting long time effects of environmental challenges, an important feature for sub-phenotyping of disease.

Since PEx is known to originate to a large extent from the small airway region we chose to include a group of asthmatic subjects with high LCI, an indirect measure of small airway involvement, and poor level of control [22]. Interestingly, we found higher number of proteins to be differentially abundant when comparing the non-asthma group including asthmatics with small airway involvement than with those without small airway involvement, indicating that PEx samples may reflect pathology that drive a more severe type of asthma, also supported by higher ACQ in that group.

The present pilot study was small and primarily dimensioned to highlight the potential of PExA as a non-invasive method for collecting small airway samples compatible with protein biomarker analysis. The fact that as many as 9 of 207 proteins were found to be differentially abundant and that all of these 9 proteins previously have been associated with pathways or mechanism that play crucial role in pulmonary disease, indicate that the proximal sampling method we used have the potential to generate a higher share of highly relevant data than what usually is expected from biomarker discovery based on blood samples.

## Conclusion

Our data illustrate for the first time how non-invasively retrieved respiratory tract lining fluid, originating from the small airways in specific, can be analyzed with regard to the relative quantity of over 150 individual proteins. Data reveal that proteins present in PEx to a large extent seem to originate from extracellular vesicles whereof many associated with innate immunity including the complement and coagulation system. Pathological relevance of PEx samples was further demonstrated by showing that all of the proteins found to be differently abundant in asthmatic subjects with small airway involvement are previously described to be involved in lung disease pathways. Collectively the results indicates that the PExA method provide a novel and non-invasive route to identify novel biomarkers and drug targets contributing to further development of precision medicine in the field of respiratory medicine.

## Supplementary Information

**Additional file 1: Table S1.** Proteins considered being detected in PEx samples using the SOMAscan 1.3 K platform. **Table S2.** Result from differential abundance analysis. **Table S3.** Result from manual reviewing the scientific literature on proteins found to be differentially abundant in A-hLCI as compared to NA group. **Table S4.** Demographic and clinical characteristics of groups based on smoking history.

## Data Availability

A list of all proteins considered detected in this study can be found under Supplementary Information.

## Abbreviations

A-nLCI: Asthma-normal lung clearance index
RFU: Relative Fluorescence Units
LOD: Limit of detection
GLM: General linear model statistics
ANOVA: Analysis of variance
PCA: Principal component analysis
GLP: Good laboratory practice
PExA: Particles in Exhaled Air, i.e. the method or instrument
PEx: The sample or biological material collected with the PExA method
LC/MS: Liquid Chromatography–Mass Spectrometry
ELISA: Enzyme-linked immunosorbent assay
EF: Enrichment Factor resulting from Gene Ontology enrichment analysis
mHG: Minimal hypergeometric model
HG: Hypergeometric model
