# Correction to: A novel non‑invasive method allowing for discovery of pathologically relevant proteins from small airways

**DOI:** 10.1186/s12014-022-09363-z

**Published:** 2022-07-18

**Authors:** Jörgen Östling, Marleen Van Geest, Henric K. Olsson, Sven-Erik Dahlen, Emilia Viklund, Per M. Gustafsson, Ekaterina Mirgorodskaya, Anna-Carin Olin

**Affiliations:** 1 Department of Bioscience, Respiratory, Infammation and Autoimmunity, IMED Biotech Unit, AstraZeneca, Gothenburg, Sweden; 2 Translational Science and Experimental Medicine, Research and Early Development, Respiratory & Immunology, BioPharmaceuticals R&D, AstraZeneca, Gothenburg, Sweden; 3 Institute of Environmental Medicine, Karolinska Institute, Stockholm, Sweden; 4 Occupational and Environmental Medicine, Department of Public Health and Community Medicine, Institute of Medicine, Sahlgrenska Academy, University of Gothenburg, Gothenburg, Sweden; 5 Department of Paediatrics, Central Hospital, Skövde, Sweden; 6 Core Facilities, Sahlgrenska Academy, University of Gothenburg, Gothenburg, Sweden; 7 PExA AB, Gothenburg, Sweden; 8 Hansa Biopharma AB, Lund, Sweden

## Correction to: Clinical Proteomics (2022) 19:20 10.1186/s12014-022-09348-y

Unfortunately, in the original version of the article, several errors were identified which were mentioned below.

In Abstract section, 1st paragraph, the sentence that reads as “These particles can be collected by impaction using the PExA^®^ … small airways” should read as “These particles can be collected by impaction using the PExA … small airways”.

In Introduction section, 6th paragraph, the sentence that reads as “The small airway origin of the PExA … detected by LC/MS [20]” should read as “The small airway origin of the PEx … detected by LC/MS [20].

In Discussion section, 7th paragraph, the sentence that reads as “The present pilot study was small and primarily dimensioned to highlight the potential of PEx … biomarker analysis” should read as “The present pilot study was small and primarily dimensioned to highlight the potential of PExA … biomarker analysis.”

The wrong Additional file was originally published with this article; it has now been replaced with the correct file.

The “Availability of data and materials” statement was incorrect in the original version and the corrected statement should read as below.

- Availability of data and materials
- A list of all proteins considered detected in this study can be found under Supplementary Information.

The original article [1] has been corrected.

## Supplementary Information

**Additional file 1.** **Table S1.** Proteins considered being detected in PExsamples using the SOMAscan 1.3 K platform. **Table S2.** Result from differentialabundance analysis. **Table S3.** Result from manual reviewingthe scientific literature on proteins found to be differentially abundantin A-hLCI as compared to NA group. **Table S4.** Demographic and clinicalcharacteristics of groups based on smoking history.
