# Supplementary material for: A novel non-invasive method allowing for discovery of pathologically relevant proteins from small airways
Source: Clin Proteomics. 2022 Jun 6;19:20. doi: 10.1186/s12014-022-09348-y (PMC9167914; doi:10.1186/s12014-022-09348-y)
Supplement: Supplementary file 1 — Additional file 1: Table S1. Proteins considered being detected in PEx samples using the SOMAscan 1.3 K platform. Table S2. Result from differential abundance analysis. Table S3. Result from manual reviewing the scientific literature on proteins found to be differentially abundant in A-hLCI as compared to NA group. Table S4. Demographic and clinical characteristics of groups based on smoking history. [file 12014_2022_9348_MOESM1_ESM.pdf]

Supplementary material

A novel non-invasive method allowing for discovery of pathologically relevant proteins from small airways.

Clinical Proteomics, Östling *et. al.* 2022

PExA sampling method

The PExA method and PExA 1.0 instrument herein used have previously been described in detail [1]. In short, the PExA 1.0 instrument report amount of PEx in real-time and counts exhaled particles in seven diameter intervals between 0.41 and 4.55 µm, (Grimm model 1.108; Grimm Aerosol Technik GmbH & Co., Ainring, Germany) and sample them by impaction (Dekati Ltd., Tampere, Finland) on a removable substrate made of hydrophilic PTFE-membrane (Millipore FHLC02500, Merck, Darmstadt, Germany). The exhalation flow and volume is monitored using an ultrasonic flow meter (OEM flow sensor; Spiroson-AS, Medical Technologies, Zürich, Switzerland).

Subjects were asked to perform a standardized breathing manoeuvre, initiated by a full exhalation to residual volume and followed by breath-holding for five seconds, a maximal inhalation to total lung capacity and finalized by a normal exhalation to functional residual capacity.

Subjects were breathing HEPA-filtered air via a mouthpiece and a two-way, non-rebreathing valve into the instrument. To avoid contamination of particles from ambient air, the air present in the airways before sampling was diluted out by a minimum of three breaths of HEPA filtered air before sampling was initiated. All subjects wore a nose clip throughout the sampling session.

PEx sample preparation

Protein were extracted from the PEx substrate by adding sample buffer (proprietary SomaLogic SB17 buffer with addition of Tween-20 to 0.1% final concentration) to the centrifugal filter insert (Millipore Ultrafree-MC LH Centrifugal Filter) containing the PEx substrate membrane. The volume of sample buffer was adjusted to reach a final concentration of PEx at either 1 or 2 µg/ml, as stated elsewhere. After addition of sample buffer, the sample tubes were briefly vortexed and placed in a thermal rotary shaker for 1 h at 30 °C and 400 rpm. After incubation on the thermal shaker, protein extracts were retrieved in the lower part of the centrifugal filter unit by centrifugation at 10,000 ×*g* for 10 min. Protein extracts were stored at -80 °C before sent to SomaLogic (Boulder, Co, USA) for analysis. Blank samples were made by extraction of the material on PEx substrates subjected to the same sampling and sample preparation procedure as all other samples, omitting the breathing manoeuvre.

SOMAscan analysis

The SOMAscan assay (SomaLogic Inc, Boulder, USA) uses slow off-rate modified DNA aptamers (SOMAmers [2] as high affinity protein capture reagents to simultaneously quantify more than 1100 human proteins in only 150 µl of serum, plasma or equally small amounts of a variety of other biological matrices. SOMAscan allows measurement of proteins across a very broad dynamic range, from low abundant cytokines to albumin, with excellent reproducibility [3-6]. To account for systematic differences due to possible variability in final PEx concentration as well as potential pipetting errors etc, the set of detected proteins were subjected to group median based normalization and log2 transformation before statistical analysis was performed.

References

1. Larsson P, Larstad M, Bake B, Hammar O, Bredberg A, Almstrand AC, Mirgorodskaya E, Olin AC. Exhaled particles as markers of small airway inflammation in subjects with asthma. *Clin Physiol Funct Imaging* 2017: 37(5): 489-497.
2. Ngo D, Sinha S, Shen D, Kuhn EW, Keyes MJ, Shi X, Benson MD, O'Sullivan JF, Keshishian H, Farrell LA, Fifer MA, Vasan RS, Sabatine MS, Larson MG, Carr SA, Wang TJ, Gerszten RE. Aptamer-Based Proteomic Profiling Reveals Novel Candidate Biomarkers and Pathways in Cardiovascular Disease. *Circulation* 2016: 134(4): 270-285.
3. Gold L, Ayers D, Bertino J, Bock C, Bock A, Brody EN, Carter J, Dalby AB, Eaton BE, Fitzwater T, Flather D, Forbes A, Foreman T, Fowler C, Gawande B, Goss M, Gunn M, Gupta S, Halladay D, Heil J, Heilig J, Hicke B, Husar G, Janjic N, Jarvis T, Jennings S, Katilius E, Keeney TR, Kim N, Koch TH, Kraemer S, Kroiss L, Le N, Levine D, Lindsey W, Lollo B, Mayfield W, Mehan M, Mehler R, Nelson SK, Nelson M, Nieuwlandt D, Nikrad M, Ochsner U, Ostroff RM, Otis M, Parker T, Pietrasiewicz S, Resnicow DI, Rohloff J, Sanders G, Sattin S, Schneider D, Singer B, Stanton M, Sterkel A, Stewart A, Stratford S, Vaught JD, Vrkljan M, Walker JJ, Watrobka M, Waugh S, Weiss A, Wilcox SK, Wolfson A, Wolk SK, Zhang C, Zichi D. Aptamer-based multiplexed proteomic technology for biomarker discovery. *PLoS One* 2010: 5(12): e15004.
4. SomaLogic_Inc. SOMAscan Proteomic Assay Technical White Paper; 2016 12/1/2017.
5. Keeney TR, Bock C, Gold L, Kraemer S, Lollo B, Nikrad M, Stanton M, Stewart A, Vaught JD, Walker JJ. Automation of the SomaLogic Proteomics Assay: A Platform for Biomarker Discovery. *JALA: Journal of the Association for Laboratory Automation* 2009: 14(6): 360-366.
6. Kraemer S, Vaught JD, Bock C, Gold L, Katilius E, Keeney TR, Kim N, Saccomano NA, Wilcox SK, Zichi D, Sanders GM. From SOMAmer-based biomarker discovery to diagnostic and clinical applications: a SOMAmer-based, streamlined multiplex proteomic assay. *PLoS One* 2011: 6(10): e26332.

Table S1. - Proteins considered being detected in PEx samples using the SOMAscan 1.3K platform

**Target Full Name**	**Entrez Gene Symbol**	**Target, alternative name**	**SomaId**	**UniProt**
14-3-3 protein epsilon	YWHAE	14-3-3E	SL004984	P62258
6-phosphogluconate dehydrogenase, decarboxylating	PGD	6-Phosphogluconate dehydrogenase	SL000247	P52209
Adapter molecule crk	CRK	CRK	SL013240	P46108
Adhesion G protein-coupled receptor E2	ADGRE2	EMR2	SL008822	Q9UHX3
Adiponectin	ADIPOQ	Adiponectin	SL004258	Q15848
Advanced glycosylation end product-specific receptor, soluble	AGER	sRAGE	SL003680	Q15109
Afamin	AFM	Afamin	SL004742	P43652
Agouti-related protein	AGRP	ART	SL006924	O00253
Alcohol dehydrogenase [NADP(+)]	AKR1A1	AK1A1	SL008039	P14550
Alpha-1-antichymotrypsin	SERPINA3	a1-Antichymotrypsin	SL000248	P01011
Alpha-1-antichymotrypsin complex	SERPINA3	alpha-1-antichymotrypsin complex	SL018548	P01011
Alpha-1-antitrypsin	SERPINA1	a1-Antitrypsin	SL000249	P01009
Alpha-2-antiplasmin	SERPINF2	a2-Antiplasmin	SL000250	P08697
Alpha-2-HS-glycoprotein	AHSG	a2-HS-Glycoprotein	SL000251	P02765
Angiostatin	PLG	Angiostatin	SL000268	P00747
Angiotensinogen	AGT	Angiotensinogen	SL000271	P01019
Ankyrin-2	ANK2	ANK2	SL014896	Q01484
Antithrombin-III	SERPINC1	Antithrombin III	SL000272	P01008
Apolipoprotein E (isoform E2)	APOE	Apo E2	SL000277	P02649
Apolipoprotein E (isoform E3)	APOE	Apo E3	SL004668	P02649
Apolipoprotein E (isoform E4)	APOE	Apo E4	SL004669	P02649
Apolipoprotein L1	APOL1	Apo L1	SL005699	O14791
Beta-2-microglobulin	B2M	b2-Microglobulin	SL000283	P61769
Beta-Ala-His dipeptidase	CNDP1	CNDP1	SL006694	Q96KN2
C3a anaphylatoxin	C3	C3a	SL000313	P01024
C3a anaphylatoxin des Arginine	C3	C3adesArg	SL003220	P01024
cAMP-dependent protein kinase catalytic subunit alpha	PRADA	PRADA	SL010513	P17612
Carbohydrate sulfotransferase 15	CHST15	ST4S6	SL007502	Q7LFX5
Carboxypeptidase B2	CPB2	TAFI	SL004015	Q96IY4
Cathepsin G	CTSG	Cathepsin G	SL000345	P08311
Cathepsin H	CTSH	Cathepsin H	SL000346	P09668
Cathepsin Z	CTSZ	CATZ	SL008380	Q9UBR2
Cation-independent mannose-6-phosphate receptor	IGF2R	IGF-II receptor	SL003679	P11717
C-C motif chemokine 18	CCL18	PARC	SL003323	P55774
C-C motif chemokine 23	CCL23	MPIF-1	SL003302	P55773

CD166 antigen	ALCAM	ALCAM	SL003166	Q13740
CD226 antigen	CD226	CD226	SL011100	Q15762
Cell adhesion molecule-related/down-regulated by oncogenes	CDON	CDON	SL014092	Q4KMG0
Chitinase-3-like protein 1	CHI3L1	YKL-40	SL003340	P36222
CMRF35-like molecule 6	CD300C	CLM6	SL014270	Q08708
Coagulation factor IX	F9	Coagulation Factor IX	SL000357	P00740
Coagulation factor IXab	F9	Coagulation Factor IXab	SL004400	P00740
Coagulation Factor X	F10	Coagulation Factor X	SL000360	P00742
Coagulation factor Xa	F10	Coagulation Factor Xa	SL003324	P00742
Collectin-12	COLEC12	COLEC12	SL007471	Q5KU26
Complement C1q subcomponent	C1QA C1QB C1QC	C1q	SL000309	P02745, P02746, P02747
Complement C1s subcomponent	C1S	C1s	SL000311	P09871
Complement C2	C2	C2	SL002525	P06681
Complement C3	C3	C3	SL000312	P01024
Complement C3b, inactivated	C3	iC3b	SL000456	P01024
Complement C3d fragment	C3	C3d	SL003362	P01024
Complement C4	C4A C4B	C4	SL000316	P0C0L4, P0C0L5
Complement C4b	C4A C4B	C4b	SL000318	P0C0L4, P0C0L5
Complement C5	C5	C5	SL000319	P01031
Complement C5b-C6 complex	C5 C6	C5b, 6 Complex	SL000321	P01031, P13671
Complement component C1q receptor	CD93	C1QR1	SL007696	Q9NPY3
Complement component C6	C6	C6	SL000322	P13671
Complement component C7	C7	C7	SL000323	P10643
Complement component C9	C9	C9	SL000325	P02748
Complement decay-accelerating factor	CD55	DAF	SL004556	P08174
Complement factor B	CFB	Factor B	SL000414	P00751
Complement factor D	CFD	Factor D	SL003327	P00746
Complement factor H	CFH	Factor H	SL000415	P08603
Complement factor I	CFI	Factor I	SL003328	P05156
Connective tissue-activating peptide III	PPBP	CTAP-III	SL004708	P02775
Contactin-1	CNTN1	contactin-1	SL004855	Q12860
Contactin-4	CNTN4	Contactin-4	SL010454	Q8IWV2
C-type mannose receptor 2	MRC2	MRC2	SL008416	Q9UBG0
C-X-C motif chemokine 16	CXCL16	CXCL16, soluble	SL004016	Q9H2A7
Cytoskeleton-associated protein 2	CKAP2	CKAP2	SL006675	Q8WWK9
Desmocollin-2	DSC2	DSC2	SL008631	Q02487
Desmoglein-2	DSG2	Desmoglein-2	SL004857	Q14126
Dickkopf-related protein 3	DKK3	DKK3	SL009412	Q9UBP4
Ectonucleoside triphosphate diphosphohydrolase 5	ENTPD5	ENTP5	SL014028	O75356
EGF-containing fibulin-like extracellular matrix protein 1	EFEMP1	FBLN3	SL006527	Q12805
Endostatin	COL18A1	Endostatin	SL000403	P39060

Endothelial cell-selective adhesion molecule	ESAM	ESAM	SL005160	Q96AP7
Endothelin-converting enzyme 1	ECE1	Endothelin-converting enzyme 1	SL004060	P42892
Ephrin-B1	EFNB1	EFNB1	SL008614	P98172
Epidermal growth factor receptor	EGFR	ERBB1	SL002644	P00533
Eukaryotic translation initiation factor 4 gamma 2	EIF4G2	IF4G2	SL011211	P78344
Extracellular matrix protein 1	ECM1	ECM1	SL006550	Q16610
Extracellular superoxide dismutase [Cu-Zn]	SOD3	SOD3	SL003672	P08294
Fatty acid-binding protein, heart	FABP3	FABP	SL001774	P05413
Ferritin	FTH1 FTL	Ferritin	SL000420	P02794, P02792
Fetuin-B	FETUB	FETUB	SL006777	Q9UGM5
Fibroblast growth factor receptor 1	FGFR1	bFGF-R	SL003060	P11362
Fibroblast growth factor receptor 3	FGFR3	FGFR-3	SL004063	P22607
Fibronectin	FN1	Fibronectin	SL000426	P02751
Fibronectin Fragment 3	FN1	FN1.3	SL010349	P02751
Fibronectin Fragment 4	FN1	FN1.4	SL010348	P02751
Follistatin-related protein 1	FSTL1	FSTL1	SL009349	Q12841
Follistatin-related protein 3	FSTL3	FSTL3	SL009324	O95633
Fructose-bisphosphate aldolase A	ALDOA	aldolase A	SL004910	P04075
Gelsolin	GSN	Gelsolin	SL005572	P06396
Glycerol-3-phosphate dehydrogenase [NAD(+)], cytoplasmic	GPD1	GPDA	SL007151	P21695
Granulins	GRN	GRN	SL007173	P28799
Heat shock protein HSP 90-alpha/beta	HSP90AA1 HSP90AB1	HSP 90a/b	SL017612	P07900, P08238
Heat shock protein HSP 90-beta	HSP90AB1	HSP 90b	SL000454	P08238
Hemopexin	HPX	Hemopexin	SL000440	P02790
Heparin cofactor 2	SERPIND1	Heparin cofactor II	SL004466	P05546
Hepatocyte growth factor activator	HGFAC	HGFA	SL006512	Q04756
Hepatocyte growth factor receptor	MET	Met	SL000134	P08581
Hepatocyte growth factor-like protein	MST1	MSP	SL005202	P26927
HERV-H LTR-associating protein 2	HHLA2	HHLA2	SL020171	Q9UM44
Heterogeneous nuclear ribonucleoprotein A/B	HNRNPAB	hnRNP A/B	SL009791	Q99729
High affinity immunoglobulin gamma Fc receptor I	FCGR1A	FCGR1	SL010461	P12314
Histidine triad nucleotide-binding protein 1	HINT1	HINT1	SL009431	P49773
Histidine-rich glycoprotein	HRG	HRG	SL006448	P04196
Immunoglobulin G	IGHG1	IgG	SL000467	P01857
Immunoglobulin M	IGHM	IgM	SL000468	P01871
Inhibin beta A chain	INHBA	Activin A	SL001938	P08476
Inhibin beta A chain:Inhibin beta B chain heterodimer	INHBA INHBB	Activin AB	SL004837	P08476, P09529
Inorganic pyrophosphatase	PPA1	PPase	SL004914	Q15181
Insulin-like growth factor-binding protein 2	IGFBP2	IGFBP-2	SL000466	P18065
Insulin-like growth factor-binding protein 3	IGFBP3	IGFBP-3	SL000045	P17936
Insulin-like growth factor-binding protein 4	IGFBP4	IGFBP-4	SL005171	P22692

Insulin-like growth factor-binding protein 6	IGFBP6	IGFBP-6	SL005172	P24592
Inter-alpha-trypsin inhibitor heavy chain H4	ITIH4	ITI heavy chain H4	SL004739	Q14624
Intercellular adhesion molecule 1	ICAM1	sICAM-1	SL002922	P05362
Intercellular adhesion molecule 5	ICAM5	sICAM-5	SL005169	Q9UMF0
Interferon alpha/beta receptor 1	IFNAR1	IFN-a/b R1	SL004475	P17181
Interleukin-1 Receptor accessory protein	IL1RAP	IL-1 R AcP	SL004588	Q9NPH3
Interleukin-6 receptor subunit alpha	IL6R	IL-6 sRa	SL001943	P08887
Interleukin-6 receptor subunit beta	IL6ST	gp130, soluble	SL003872	P40189
Kallistatin	SERPINA4	Kallistatin	SL004876	P29622
Kininogen-1	KNG1	Kininogen, HMW	SL017189	P01042
Lactadherin	MFGE8	MFGM	SL006523	Q08431
Leucine-rich repeat transmembrane protein FLRT3	FLRT3	FLRT3	SL008372	Q9NZU0
Leukocyte immunoglobulin-like receptor subfamily B member 1	LILRB1	ILT-2	SL005190	Q8NHL6
Leukocyte immunoglobulin-like receptor subfamily B member 2	LILRB2	ILT-4	SL005191	Q8N423
Leukotriene A-4 hydrolase	LTA4H	LKHA4	SL007100	P09960
Lipopolysaccharide-binding protein	LBP	LBP	SL003309	P18428
Lithostathine-1-alpha	REG1A	PSP	SL005357	P05451
L-lactate dehydrogenase B chain	LDHB	LDH-H 1	SL000493	P07195
Low affinity immunoglobulin gamma Fc region receptor III-B	FCGR3B	FCG3B	SL008609	O75015
Low-density lipoprotein receptor-related protein 1B	LRP1B	LRP1B	SL013872	Q9NZR2
L-Selectin	SELL	sL-Selectin	SL002823	P14151
Lumican	LUM	Lumican	SL006230	P51884
Lymphocyte antigen 86	LY86	LY86	SL007059	O95711
Macrophage colony-stimulating factor 1 receptor	CSF1R	M-CSF R	SL004153	P07333
Macrophage mannose receptor 1	MRC1	Macrophage mannose receptor	SL004579	P22897
Macrophage-capping protein	CAPG	CAPG	SL008099	P40121
Metalloproteinase inhibitor 2	TIMP2	TIMP-2	SL000592	P16035
Myoglobin	MB	Myoglobin	SL000164	P02144
Neurexin-3-beta	NRXN3	NRX3B	SL008728	Q9HDB5
Neurogenic locus notch homolog protein 1	NOTCH1	Notch 1	SL005703	P46531
Neurogenic locus notch homolog protein 3	NOTCH3	Notch-3	SL005209	Q9UM47
Neuropilin-1	NRP1	NRP1	SL006397	O14786
Nucleoside diphosphate kinase B	NME2	NDP kinase B	SL004921	P22392
Phosphoglycerate kinase 1	PGK1	phosphoglycerate kinase 1	SL003653	P00558
Pigment epithelium-derived factor	SERPINF1	PEDF	SL003066	P36955
Plasma kallikrein	KLKB1	Prekallikrein	SL000545	P03952
Plasma protease C1 inhibitor	SERPING1	C1-Esterase Inhibitor	SL000308	P05155
Plasma serine protease inhibitor	SERPINA5	PCI	SL000550	P05154
Plasminogen	PLG	Plasminogen	SL000541	P00747
Plexin-B2	PLXNB2	PLXB2	SL009948	O15031
Polymeric immunoglobulin receptor	PIGR	PIGR	SL005797	P01833

Pro-opiomelanocortin	POMC	Corticotropin-lipotropin	SL009210	P01189
Properdin	CFP	Properdin	SL003192	P27918
Proprotein convertase subtilisin/kexin type 9	PCSK9	PCSK9	SL012707	Q8NBP7
Protein kinase C alpha type	PRKCA	PKC-A	SL000551	P17252
Protein Rev_HV2BE	Human-virus	HIV-2 Rev	SL000445	P18093
Prothrombin	F2	Prothrombin	SL000558	P00734
Ras GTPase-activating protein 1	RASA1	RASA1	SL013754	P20936
Ras-related C3 botulinum toxin substrate 1	RAC1	RAC1	SL004009	P63000
Repulsive guidance molecule A	RGMA	RGMA	SL010467	Q96B86
Reticulon-4	RTN4	RTN4	SL008309	Q9NQC3
Reticulon-4 receptor	RTN4R	Nogo Receptor	SL005208	Q9BZR6
Retinoic acid receptor responder protein 2	RARRES2	TIG2	SL005152	Q99969
RGM domain family member B	RGMB	RGMB	SL010468	Q6NW40
Scavenger receptor class F member 1	SCARF1	SREC-I	SL005221	Q14162
Scavenger receptor cysteine-rich type 1 protein M130	CD163	sCD163	SL005764	Q86VB7
Secreted and transmembrane protein 1	SECTM1	SECTM1	SL005430	Q8WVN6
Serine/threonine-protein kinase WNK3	WNK3	WNK3	SL010524	Q9BYP7
Serotransferrin	TF	Transferrin	SL000601	P02787
Serum albumin	ALB	Albumin	SL000254	P02768
Serum amyloid P-component	APCS	SAP	SL000573	P02743
Sex hormone-binding globulin	SHBG	SHBG	SL005102	P04278
Sialic acid-binding Ig-like lectin 14	SIGLEC14	SIG14	SL014292	Q08ET2
Sialic acid-binding Ig-like lectin 7	SIGLEC7	Siglec-7	SL005218	Q9Y286
Signal transducer and activator of transcription 3	STAT3	STAT3	SL007221	P40763
SPARC	SPARC	ON	SL000532	P09486
Stanniocalcin-1	STC1	Stanniocalcin-1	SL005789	P52823
Tartrate-resistant acid phosphatase type 5	ACP5	TrATPase	SL004118	P13686
Testican-2	SPOCK2	Testican-2	SL010471	Q92563
Thrombospondin-4	THBS4	TSP4	SL007207	P35443
Thyroxine-binding globulin	SERPINA7	Thyroxine-Binding Globulin	SL000590	P05543
Tissue factor pathway inhibitor	TFPI	TFPI	SL001998	P10646
Transforming growth factor beta receptor type 3	TGFBR3	TGF-b R III	SL005059	Q03167
Transforming growth factor-beta-induced protein ig-h3	TGFBI	BGH3	SL006544	Q15582
Transgelin-2	TAGLN2	Transgelin-2	SL004811	P37802
Tropomyosin alpha-4 chain	TPM4	Tropomyosin 4	SL003646	P67936
Tumor necrosis factor receptor superfamily member 10B	TNFRSF10B	TRAIL R2	SL004157	O14763
Tumor necrosis factor receptor superfamily member 1A	TNFRSF1A	TNF sR-I	SL001992	P19438
Tumor necrosis factor receptor superfamily member 1B	TNFRSF1B	TNF sR-II	SL001800	P20333
Tumor necrosis factor receptor superfamily member 21	TNFRSF21	DR6	SL004871	O75509
Tumor necrosis factor receptor superfamily member 25	TNFRSF25	DR3	SL004791	Q93038
Urokinase plasminogen activator surface receptor	PLAUR	suPAR	SL002506	Q03405

Vascular endothelial growth factor A, isoform 121	VEGFA	VEGF121	SL003310	P15692
Vascular endothelial growth factor D	FIGF	VEGF-D	SL003320	O43915
Vitamin K-dependent protein C	PROC	Protein C	SL000048	P04070
Vitamin K-dependent protein S	PROS1	Protein S	SL000049	P07225
Vitronectin	VTN	Vitronectin	SL000616	P04004
von Willebrand factor	VWF	vWF	SL000017	P04275
Wnt inhibitory factor 1	WIF1	WIF-1	SL004652	Q9Y5W5
WNT1-inducible-signaling pathway protein 1	WISP1	WISP-1	SL004689	O95388

Names of the 207 proteins that were detected with SOMAscan 1.3K platform, in at least 80% of 30 PEx samples used for assessment of pathological relevance of proteins detected in PEx samples, as described in Material and Methods.

Table S2. - Result from differential abundance analysis

Target Full Name	Entrez Gene Symbol	A-hLCI vs. NA			A_hLCI vs. A-nLCI			A-hLCI vs. A-nLCI + NA		
		p-value	q-value	Fold change	p-value	q-value	Fold change	p-value	q-value	Fold change
Vascular endothelial growth factor D	FIGF	0.0003	0.069	1.184	n.s	n.s	1.099	0.001	0.038	1.147
Alpha-1-antitrypsin	SERPINA1	0.0008	0.083	1.182	0.002	n.s	1.283	0.001	0.038	1.202
Complement component C1q receptor	CD93	0.0017	0.120	1.320	0.017	n.s	1.291	0.001	0.038	1.286
C-C motif chemokine 18	CCL18	0.0034	0.152	1.917	n.s	n.s	1.457	0.021	0.167	1.718
Fructose-bisphosphate aldolase A	ALDOA	0.0037	0.152	0.631	0.018	n.s	0.615	0.002	0.040	0.650
Complement C4	C4A, C4B	0.0052	0.170	0.633	n.s.	n.s	0.704	0.002	0.043	0.660
Coagulation Factor X	F10	0.0068	0.170	1.305	0.038	n.s	1.265	0.007	0.092	1.277
Immunoglobulin M	IGHM	0.0070	0.170	1.216	0.007	n.s	1.303	0.001	0.038	1.247
Interleukin-1 Receptor accessory protein	IL1RAP	0.0074	0.170	1.260	0.020	n.s	1.292	0.001	0.038	1.275

Statistics for proteins found to be differentially abundant when comparing SOMAscan data for 207 proteins between the Asthma with high LCI (A-hLCI, n=10) and Non-asthma control group (NA, n=10), using a significance criteria including p-value below 0.05 and Benjamini–Hochberg corrected p-value (q) below 0.2, as described in Material and Methods. Statistics for comparison with asthma with normal LCI (A-nLCI) is also shown. Significance values above the threshold are indicated with n.s. Fold change was defined as 2 to the power of the difference difference being defined as the difference of the mean log2 of "group1" data and mean log2 of "group2" data. Fold change values >1 and < 1 indicate higher and lower levels in the first mentioned group, respectively. No significant difference were found for the A-nLCI vs. NA group comparison. Uniprot protein IDs and synonyms can be found in Table S1A

Table S3.

Result from manual reviewing the scientific literature on proteins found to be differentially abundant in A-hLCI as compared to NA group.

**Protein name**<br>Entrez symbol and synonyms<br>(Uniprot ID)	**Abundance profile**[#]	**Relevant literature findings, summary and reference**
**Alpha-1-antitrypsin**<br>SERPINA1<br>A1AT<br>(P01009)	NA L<br>A-nLCI L<br>A-hLCI **H**	- Found to be the major inhibitor of neutrophil elastase in the lower respiratory system [1, 2].<br>- Associated with an increased risk of pulmonary and extrapulmonary disease [3].<br>- Protects **alveoli** against the destructive effects of neutrophil elastase, proteinase 3 and cathepsin G [4].<br>- Increased levels in circulation is associated with pathology localised to the **Small airway** and alveolar space [5].
**Interleukin-1 receptor accessory protein**<br>IL1RAP<br>IL1R3<br>IL-1 R AcP<br>(Q9NPH3)	NA L<br>A-nLCI L<br>A-hLCI **H**	- Localized to the cytosol & vesicles. Involved in e.g, immune and inflammatory response, innate immune response, IL-1 and IL-33-mediated signalling (GO annotations) [6, 7].<br>- Soluble isoform exist (sIL1RAP) [8].<br>- Elevated levels is associated with reduced FEV1/FVC. Suggested to be a marker and potential therapeutic target in patients with **neutrophilic asthma** and airflow obstruction [9].<br>- Elevated in patients with COPD [10].<br>- Is required for IL-33 signalling which is important in promoting and maintaining the **asthma** phenotype [11].
**C-C motif chemokine 18**<br>CCL18<br>(P55774)	NA L<br>A-nLCI IM<br>A-hLCI **H**	- mRNA particularly abundant in lung (Protein Atlas) [12, 13]<br>- Levels in serum, BALF and Alveolar macrophage culture supernatant are markedly increased in various interstitial **lung diseases** [14].<br>- Suggested to play a predominant role in allergic **asthma** [15, 16].<br>- Suggested to serve as a circulating biomarker in non-small cell lung cancer diagnosis [17].
**Complement component C1q receptor**<br>CD93<br>C1QR1<br>(Q9NPY3)	NA L<br>A-nLCI IM<br>A-hLCI **H**	- mRNA and protein particularly abundant in lung (Protein Atlas) [12, 13].<br>- Involved in e.g. macrophage activation and **neutrophil** degranulation (GO annotations) [6, 7].<br>- Have been observed to be expressed at high levels in lung and at elevated levels in circulation of **asthmatics**.<br>- Have been suggested as a circulating biomarker with potential to aid in **asthma** diagnosis [18, 19].

**IgM**<br>IGHM<br>(P01871)	NA L<br>A-nLCI IM<br>A-hLCI **H**	- Involved in innate immune response & complement activation, classical pathway (GO annotations) [6, 7].<br>- Play role in host defence, immune regulation and immunological tolerance. Locally **synthetized in airways**.<br>- Elevated levels have been observed in **RTLF** from **asthmatic** subjects. Suggested to play role in pathogenesis of **asthma**. [20, 21].
**Vascular endothelial growth factor D**<br>FIGF<br>VEGF-D<br>(O43915)	NA L<br>A-nLCI IM<br>A-hLCI **H**	- Localized to the extracellular space, platelet alpha granule lumen. Involved in platelet degranulation (GO annotations) [6, 7].<br>- Contributes to the **small-airway** remodelling in a rat model of COPD [22].<br>- Increased levels of vascular endothelial growth factor in induced sputum of asthmatics.. Suggested to play important role in the pathogenesis of bronchial **asthma** [23].
**Coagulation Factor X**<br>FX<br>F10<br>(P00742)	NA L<br>A-nLCI IM<br>A-hLCI **H**	- Uncontrolled activation of the coagulation cascade contributes to the pathophysiology of several conditions, including acute and chronic lung diseases. Protease-activated receptors have been implicated as the molecular link between coagulation and allergic inflammation in **asthma** [24].<br>- In contradiction with our finding showing lower levels of FX in PEx from asthmatic subjects as compared healthy controls, some studies report increased levels of FX in BALF. However, other studies report similar result with significantly decreased levels in airways in **severe asthma** and intermediate levels in **moderate asthma**, as compared to healthy controls [25].
**Fructose-bisphosphate aldolase A**<br>ALDOA<br>ALDA<br>(P04075)	NA **H**<br>A-nLCI IM<br>A-hLCI L	- Localized to the extracellular region, platelet alpha granule lumen, extracellular exosome. Involved in platelet degranulation, neutrophil degranulation (GO annotations) [6, 7].<br>- Patients with asthma have high presence of nitrotyrosine in both the airways and the lung parenchyma [25].<br>- Has been observed to undergo post-translational regulation by protein tyrosine nitration in mast cells. Suggested to be an important pathway that regulates mast cell phenotype and function [26-28].<br>- Nitrotyrosine formation in airway epithelial and inflammatory cells is elevated in asthma and COPD [37].<br>- The ability to detect post-translational modifications is an important advantage of aptamers as tools for identification and detection of biomarkers, as exemplified by Ray. P et al [29]. The lower levels of ALDOA in A-hLCI group may be explained by tyrosine nitration of ALDOA proteins in mast cells in the A-hLCI group, resulting in weaker binding of the SOMAscan aptamer to the nitrated isoform of ALDOA, giving rise to weaker signal in A-hLCI group.
**Complement C4**<br>C4A<br>(P0C0L4)	NA **H**<br>A-nLCI IM<br>A-hLCI L	- Localized to extracellular region, blood microparticle. Involved in innate immune response, inflammatory response, complement activation (GO annotations) [6, 7].<br>- Pulmonary **alveolar type II epithelial cells** synthesize and secrete complement proteins C2, C3, C4, C5, and Factor B. Studies have demonstrated that complement may serve as a key link between innate and adaptive immunity in a **variety of pulmonary conditions** [30].

Footnote; Abundance profile indicate the level of the protein in PEx in one group in relation to the other groups; Non-Asthma (NA), Asthma with normal LCI (A-nLCI), Asthma with high LCI (A-hLCI), higher level (H), intermediate level (IM), lower level (L). Reference in brackets refer to references below and not to references in the main article.

References specific for Table S3

1. Gadek JE, Fells GA, Zimmerman RL, Rennard SI, Crystal RG. Antielastases of the human alveolar structures. Implications for the protease-antiprotease theory of emphysema. The Journal of Clinical Investigation 1981: 68(4): 889-898.
2. Janoff A. Elastases and emphysema. Current assessment of the protease-antiprotease hypothesis. Am Rev Respir Dis 1985: 132(2): 417-433.
3. da Costa Dias de Sousa C, Meira L, Sucena M. Alpha-1 antitrypsin deficiency – a potentially fatal disease. European Respiratory Journal 2017: 50(suppl 61): PA4463.
4. Suárez-Lorenzo I, de Castro FR, Cruz-Niesvaara D, Herrera-Ramos E, Rodríguez-Gallego C, Carrillo-Diaz T. Alpha 1 antitrypsin distribution in an allergic asthmatic population sensitized to house dust mites. Clinical and Translational Allergy 2018: 8(1): 44.
5. Welle I, Bakke PS, Eide GE, Fagerhol MK, Omenaas E, Gulsvik A. Increased circulating levels of α₁-antitrypsin and calprotectin are associated with reduced gas diffusion in the lungs. European Respiratory Journal 2001: 17(6): 1105-1111.
6. Consortium TGO. The Gene Ontology Resource: 20 years and still GOing strong. Nucleic Acids Res 2019: 47(D1): D330-d338.
7. Ashburner M, Ball CA, Blake JA, Botstein D, Butler H, Cherry JM, Davis AP, Dolinski K, Dwight SS, Eppig JT, Harris MA, Hill DP, Issel-Tarver L, Kasarskis A, Lewis S, Matese JC, Richardson JE, Ringwald M, Rubin GM, Sherlock G. Gene ontology: tool for the unification of biology. The Gene Ontology Consortium. Nat Genet 2000: 25(1): 25-29.
8. Smith DE, Hanna R, Della F, Moore H, Chen H, Farese AM, MacVittie TJ, Virca GD, Sims JE. The soluble form of IL-1 receptor accessory protein enhances the ability of soluble type II IL-1 receptor to inhibit IL-1 action. Immunity 2003: 18(1): 87-96.
9. Evans MD, Esnault S, Denlinger LC, Jarjour NN. Sputum cell IL-1 receptor expression level is a marker of airway neutrophilia and airflow obstruction in asthmatic patients. The Journal of allergy and clinical immunology 2018: 142(2): 415-423.
10. Xia J, Zhao J, Shang J, Li M, Zeng Z, Zhao J, Wang J, Xu Y, Xie J. Increased IL-33 expression in chronic obstructive pulmonary disease. Am J Physiol Lung Cell Mol Physiol 2015: 308(7): L619-627.
11. Palmer G, Lipsky BP, Smithgall MD, Meininger D, Siu S, Talabot-Ayer D, Gabay C, Smith DE. The IL-1 receptor accessory protein (AcP) is required for IL-33 signaling and soluble AcP enhances the ability of soluble ST2 to inhibit IL-33. Cytokine 2008: 42(3): 358-364.
12. Human Protein Atlas. [cited; June, 2020:[Web site, database]. Available from: http://www.proteinatlas.org
13. Uhlen M, Fagerberg L, Hallstrom BM, Lindskog C, Oksvold P, Mardinoglu A, Sivertsson A, Kampf C, Sjostedt E, Asplund A, Olsson I, Edlund K, Lundberg E, Navani S, Szigyarto CA, Odeberg J, Djureinovic D, Takanen JO, Hober S, Alm T, Edqvist PH, Berling H, Tegel H, Mulder J, Rockberg J, Nilsson P, Schwenk JM, Hamsten M, von Feilitzen K, Forsberg M, Persson L, Johansson F, Zwahlen M, von Heijne G, Nielsen J, Ponten F. Proteomics. Tissue-based map of the human proteome. Science 2015: 347(6220): 1260419.
14. Cai M, Bonella F, He X, Sixt SU, Sarria R, Guzman J, Costabel U. CCL18 in serum, BAL fluid and alveolar macrophage culture supernatant in interstitial lung diseases. Respir Med 2013: 107(9): 1444-1452.

15. de Nadai P, Charbonnier AS, Chenivesse C, Senechal S, Fournier C, Gilet J, Vorng H, Chang Y, Gosset P, Wallaert B, Tonnel AB, Lassalle P, Tsicopoulos A. Involvement of CCL18 in allergic asthma. J Immunol 2006: 176(10): 6286-6293.
16. Tsicopoulos A, Chang Y, Ait Yahia S, de Nadai P, Chenivesse C. Role of CCL18 in asthma and lung immunity. Clin Exp Allergy 2013: 43(7): 716-722.
17. Huang H, Li J, Hu W-j, Chen C, Luo H-q, Tang X-d, Zhou K-y, Zhong W-t, Li X-y. The serum level of CC chemokine ligand 18 correlates with the prognosis of non-small cell lung cancer. The International Journal of Biological Markers 2019: 34(2): 156-162.
18. Park HJ, Oh E-Y, Han H-J, Park KH, Jeong K-Y, Park J-W, Lee J-H. Potential Role of Soluble CD93 in Allergic Asthma. Journal of Allergy and Clinical Immunology 2019: 143(2): AB216.
19. Park HJ, Oh E-Y, Han H-J, Park KH, Jeong K-Y, Park J-W, Lee J-H. Soluble CD93 in allergic asthma. Scientific Reports 2020: 10(1): 323.
20. Hol BE, van de Graaf EA, Out TA, Hische EA, Jansen HM. IgM in the airways of asthma patients. Int Arch Allergy Appl Immunol 1991: 96(1): 12-18.
21. Peebles RS, Jr., Liu MC, Lichtenstein LM, Hamilton RG. IgA, IgG and IgM quantification in bronchoalveolar lavage fluids from allergic rhinitics, allergic asthmatics, and normal subjects by monoclonal antibody-based immunoenzymetric assays. J Immunol Methods 1995: 179(1): 77-86.
22. Wang L, Xu Z, Chen B, He W, Hu J, Zhang L, Liu X, Chen F. The Role of Vascular Endothelial Growth Factor in Small-airway Remodelling in a Rat Model of Chronic Obstructive Pulmonary Disease. Scientific Reports 2017: 7(1): 41202.
23. Asai K, Kanazawa H, Kamoi H, Shiraishi S, Hirata K, Yoshikawa J. Increased levels of vascular endothelial growth factor in induced sputum in asthmatic patients. Clin Exp Allergy 2003: 33(5): 595-599.
24. de Boer JD, Majoor CJ, van 't Veer C, Bel EHD, van der Poll T. Asthma and coagulation. Blood 2012: 119(14): 3236-3244.
25. Sergei A. Kharitonov KI. Nitric Oxide. In: Peter J. Barnes JMD, Stephen I. Rennard, Neil C. Thomson, ed. Asthma and COPD: Basic Mechanisms and Clinical Management. Elsevier. , 2009; pp. 363-365.
26. Pejler G. The emerging role of mast cell proteases in asthma. European Respiratory Journal 2019: 1900685.
27. Sekar Y, Chul Moon T, Slupsky CM, Befus AD. Effects of nitric oxide-induced aldolase nitration on the glycolytic metabolites of mast cell. Allergy, Asthma & Clinical Immunology 2010: 6(1): P21.
28. Sekar Y, Moon TC, Slupsky CM, Befus AD. Protein tyrosine nitration of aldolase in mast cells: a plausible pathway in nitric oxide-mediated regulation of mast cell function. J Immunol 2010: 185(1): 578-587.
29. Ray P, Sullenger BA, White RR. Further characterization of the target of a potential aptamer biomarker for pancreatic cancer: cyclophilin B and its posttranslational modifications. Nucleic Acid Ther 2013: 23(6): 435-442.
30. Pandya PH, Wilkes DS. Complement system in lung disease. Am J Respir Cell Mol Biol 2014: 51(4): 467-473.

Table S4. Demographic and clinical characteristics of groups based on smoking history

Parameter	Nevers smokers	Ex-smokers
Number	16	14
Asthma y/n	7/9	11/3
Packyear	-	3.36 (1.29) [0-12]
Gender (Male/Female)	8/8	8/6
Age	40.1 (3.38) [20-63]	52.4 (3.5) [28-68]
Age at onset of asthma, yrs	-	17.4 (5.16) [5-55]
BMI	24.9 (0.73) [19.3-31.2]	24.7 (0.7) [21.2-29.1]
Allergy y/n	11/5	8/6
FEV1 (% pred)	91.6 (3.05) [77-117]	82.6 (6.13) [39-123]
FEV1/FVC (%)	74.3 (4.84) [7.8-93.8]	69.2 (3.74) [35-88]
Reversibility (%)	8.1 (2.08) [-5-24]	9.7 (2.24) [2-28]
ACQ, mean (1-6) >1 (y/n)	3/4	5/5
B-neutrophils (%)	3.26 (0.27) [1.5-5.4]	4.37 (0.4) [2.2-7.3]
B-eosinophils (%)	0.22 (0.04) [0.06-0.6]	0.29 (0.05) [0.1-0.6]
FENO, ppb	39.64 (7.13) [6-113]	30 (7.7) [10-103]
hsCRP (n=29)	0.81 (0.26) [0.14-4.2]	1.47 (0.48) [0.14-5.4] (n=13)
Average mass pg/particle	0.21 (0.01) [0.18-0.29]	0.23 (0.01) [0.17-0.3]

Data are presented as means with standard error given in parenthesis and range given in brackets. Incomplete data is indicated by n-number given in parenthesis. Dash (-) indicate "not applicable"
